# NF-κB inhibition by dimethylaminoparthenolide radiosensitizes non-small-cell lung carcinoma by blocking DNA double-strand break repair

**DOI:** 10.1038/s41420-017-0008-3

**Published:** 2018-02-07

**Authors:** Peter V. Deraska, Colin O’Leary, Hunter D. Reavis, Shelby Labe, Tru-Khang Dinh, Jean-Bernard Lazaro, Christopher Sweeney, Alan D. D’Andrea, David Kozono

**Affiliations:** 1 Department of Radiation Oncology, Dana-Farber Cancer Institute, Boston, MA USA; 2 Center for DNA Damage and Repair, Dana-Farber Cancer Institute, Boston, MA USA; 3 Department of Medical Oncology, Dana-Farber Cancer Institute, Boston, MA USA; 4 Department of Pediatric Oncology, Dana-Farber Cancer Institute, Boston, MA USA

## Abstract

Despite optimal chemotherapy, radiotherapy (RT), and/or surgery, non-small-cell lung carcinoma (NSCLC) remains the leading cause of cancer-related death in the US and worldwide. Thoracic RT, a mainstay in the treatment of locally advanced NSCLC, is often restricted in efficacy by a therapeutic index limited by sensitivity of tissues surrounding the malignancy. Therefore, radiosensitizers that can improve the therapeutic index are a vital unmet need. Inhibition of the NF-κB pathway is a proposed mechanism of radiosensitization. Here we demonstrate that inhibition of the canonical NF-κB pathway by dimethylaminoparthenolide (DMAPT) radiosensitizes NSCLC by blocking DNA double-strand break (DSB) repair. NF-κB inhibition results in significant impairment of both homologous recombination (HR) and non-homologous end joining (NHEJ), as well as reductions in ionizing radiation (IR)-induced DNA repair biomarkers. NF-κB inhibition by DMAPT shows preclinical potential for further investigation as a NSCLC radiosensitizer.

## Introduction

Radiation therapy (RT) is a critical modality in the treatment of locally advanced, e.g., Stage III, non-small-cell lung carcinoma (NSCLC), especially when the tumor is not surgically resectable^1,2^. However, a limitation in locally advanced NSCLC therapy is our inability to treat the tumor to a sufficient dose of radiation to achieve consistently high rates of locoregional control. This is due to anticipated toxicities in surrounding radiosensitive structures including the heart, spinal cord, esophagus, and uninvolved lung^3–6^. Efforts to increase the radiation dose from 60 to 74 Gy resulted in higher levels of radiation-induced toxicity, an increase in locoregional failure and a decrease in overall survival^7^. As increased radiation doses cannot be used, efforts to improve RT efficacy require alterations in the therapeutic index. Radiosensitizing agents, beyond cytotoxic chemotherapeutics, may address this unmet need, and thus targeted therapies that may synergize with RT have garnered interest^8^.

DNA double-strand breaks (DSBs) are among the most characteristic and toxic lesions created by RT^9^. Upon induction of a DNA DSB, the cell has multiple pathways by which the lesion can be repaired, with homologous recombination (HR) and non-homologous end joining (NHEJ) being the predominant repair pathways^10^. NSCLC is characterized as a relatively HR proficient malignancy. Analysis of patient samples indicated that HR genes, including BRCA1 and BRCA2, are upregulated in NSCLC^11^. Furthermore, a study of human NSCLC tumor biopsies and cell lines revealed that most samples formed RAD51 foci and were resistant to standard DNA-damaging therapies, suggesting HR proficiency^12^. HR proficiency has been associated with radioresistance, making the HR pathway a promising target for radiosensitization in NSCLC^13^. Likewise, it has been suggested that NHEJ is the mechanism by which the majority of ionizing radiation (IR)-induced DNA DSBs are repaired^14^. Studies on small molecules that directly (DNA-PK inhibitors) and indirectly (EGFR inhibitors) impair NHEJ indicated that NHEJ inhibition increases NSCLC radiosensitivity^15,16^. Therefore, developing additional small molecules that impair DNA DSB repair pathways may result in new radiosensitizers that can be used to treat NSCLC.

The NF-κB pathway has known roles in therapeutic resistance to IR and other DNA-damaging agents^17–19^. Following exposure to genotoxic stresses, cells activate the NF-κB pathway in an ATM-dependent manner, leading to the degradation of the cytoplasmic sequestering protein IκBα and translocation of NF-κB to the nucleus^20^. Canonically, the NF-κB pathway is widely considered to mitigate the response to genotoxic stress by promoting the transcription of several anti-apoptotic genes. In addition, NF-κB regulates the expression of several fanconi anemia (FA)/HR genes involved in DNA DSB repair^21,22^. Previous work demonstrated that proteasome inhibitors, which are known to impair NF-κB signaling, increase sensitivity to DNA-damaging agents, including IR, in vitro by impairing the NF-κB-mediated expression of FA/HR genes^22,23^. As such, NF-κB pathway inhibition appears to be a promising strategy for radiosensitization in NSCLC. Proteasome inhibitors, however, have effects on wide-ranging pathways besides NF-κB^24^. We therefore examined whether a more specific small-molecule NF-κB inhibitor may be effective in radiosensitizing NSCLC by abrogating the DNA damage response following IR.

DMAPT is a water-soluble and orally bioavailable analog of the feverfew plant derivative parthenolide with activity against the NF-κB pathway^25^. Studies on NSCLC tumor xenograft models demonstrated that DMAPT is bioavailable in vivo^26,27^. Previous studies demonstrated that DMAPT radiosensitizes NSCLC and impairs DNA DSB repair, potentially by invoking a cell cycle defect^26^. Other studies have proposed that DMAPT cancer cytotoxicity may be due to NF-κB-independent mechanisms, including reactive oxygen species (ROS) accumulation via glutathione depletion and c-Jun inactivation^28–30^. While these effects may be important in other settings, we hypothesized that NSCLC radiosensitization is due to the direct effect of DMAPT on NF-κB inhibition. Here we demonstrate that DMAPT inhibits both HR and NHEJ, using reporter assays for these pathways, and explore mechanisms by which it does so in a cell cycle-independent and NF-κB-dependent manner.

## Results

### DMAPT inhibits NF-κB pathway activity and abrogates NF-κB response to IR in a cell cycle-independent manner

**Fig. 1:**
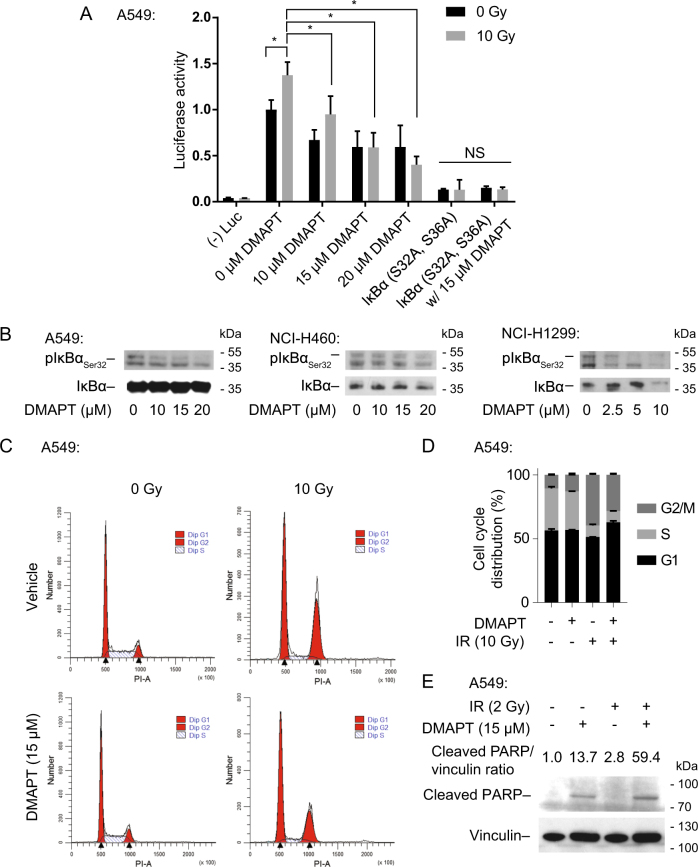
DMAPT inhibits NF-κB pathway activity in a cell cycle-independent manner and abrogates NF-κB response to IR **a** Luciferase NF-κB activity assay in A549 cells treated with a range of DMAPT doses with or without 10 Gy ionizing radiation (IR). Cells were transfected with a non-phosphorylatable IκBα construct (NF-κB super-repressor (SR)) to inhibit NF-κB signaling as a positive control. Bars show mean ± SD (*n* = 3). * indicates *p* < 0.05 by Tukey multiple comparison test. **b** Western blot showing IκBα Ser32 phosphorylation levels following DMAPT treatment in A549, NCI-H460, and NCI-H1299 cells. Cells were treated with DMAPT for 24 h and bortezomib for 1 h prior to harvest; bortezomib was added to permit visualization of the otherwise rapidly degraded protein. **c** Cell cycle analysis of A549 cells following DMAPT treatment and/or 10 Gy IR. Cells were harvested 6 h following IR or incubation without IR and analyzed by propidium iodide staining. **d** Quantification of **c**. Bars show mean ± SD (*n* = 3). **e** Western immunoblotting for cleaved PARP in A549 cells treated with 2 Gy IR and/or 15 µM DMAPT to assess induction of apoptosis

We performed the following assays in NSCLC cell lines whose viability was reduced by DMAPT throughout a range of concentrations (Supplementary Fig. 1). While DMAPT has been characterized by its action on multiple pathways including ROS and c-Jun^28–30^, the effect of parthenolide on NF-κB has been shown to be independent of p38 and c-Jun activation^31^. NF-κB inhibition by DMAPT was confirmed using a transcriptional NF-κB luciferase reporter assay (Fig. 1a). DMAPT decreased NF-κB activity in a dose-dependent manner and abrogated 10 Gy IR-induced NF-κB activity. DMAPT did not further decrease NF-κB activity in cells expressing a non-phosphorylatable IκBα mutant NF-κB super-repressor^32^, suggesting specificity of the effect of DMAPT on IκBα. To further confirm the mechanism of NF-κB inhibition, IκB kinase (IKK)-mediated IκBα Ser32 phosphorylation, a necessary step for activation of the canonical NF-κB pathway^33^, was assessed. To observe levels of phosphorylated IκBα, which is rapidly degraded following phosphorylation^33^, bortezomib was used to inhibit its proteasomal degradation (Supplementary Fig. S2A). Under these conditions, treatment of NSCLC cells with increasing concentrations of DMAPT showed dose-dependent decreases in pIκBα_Ser32_ levels consistent with inhibition of IKK (Fig. 1b). Specificity of the pIκBα_Ser32_ antibody was confirmed by siRNA knockdown (Supplementary Fig. S2B).

DMAPT has been previously shown to impact the cell cycle^27^. Because several DNA repair genes, including those involved in HR-mediated DSB repair, are cell cycle regulated^34^, we explored induction of a cell cycle defect as a potential mechanism of radiosensitization. However, cell cycle profiling of three NSCLC cell lines did not show alterations induced by DMAPT alone (Fig. 1c, d; Supplementary Fig. S3). IR showed an expected increase in percentage of cells in G2/M^35^, while the addition of DMAPT partially reversed this increase. Since G2 and M are generally held to be the most radiosensitive phases of the cell cycle^36^, if anything this observation goes counter to the hypothesis that radiosensitization by DMAPT is due to its impact on the cell cycle. To assess the mechanism of reduced cell viability following DMAPT and/or IR treatment, apoptosis was assessed by western blotting for cleaved PARP. DMAPT-induced apoptosis was increased by combination with IR (Fig. 1e).

### NF-κB inhibition by DMAPT sensitizes NSCLC cells to IR by inhibiting repair of DNA DSBs

**Fig. 2:**
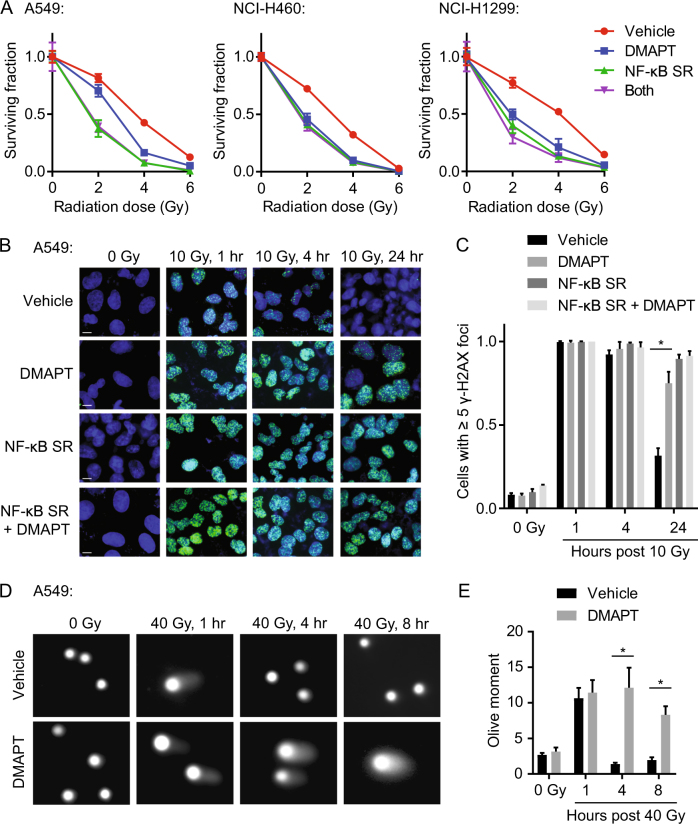
NF-κB inhibition by DMAPT sensitizes NSCLC cells to IR in vitro by inhibiting repair of DNA DSBs **a** Clonogenic survival assays in A549, NCI-H460, and NCI-H1299 cells following treatment with or without DMAPT and IR. Cells were transfected with an NF-κB super-repressor (SR) to inhibit the NF-κB pathway prior to treatment to assess for NF-κB-independent effects. All points show mean ± SD (*n* = 3). There were statistically significant differences (*p* < 0.05) in the surviving fractions of cells treated with 2 or 4 Gy with vs. without DMAPT in all three cell lines. There were no statistically significant differences in the surviving fraction of cells expressing the NF-κB SR with vs. without DMAPT treatment. See Supplementary Fig. S4 for statistical inference of synergy. **b** Immunofluorescence of γ-H2AX foci in A549 cells treated with DMAPT and/or IR over a 24-h period post IR. Cells were transfected with an NF-κB super-repressor (SR) to inhibit the NF-κB pathway prior to treatment to assess for NF-κB-independent effects. (blue: DAPI, green: γ-H2AX). Scale bar = 10 µm. **c** Quantification of **b**. Bars show mean ± SD (*n* = 3). * indicates *p* < 0.05 by *t*-test. **d** Comet assay images of A549 cells treated with DMAPT and/or 40 Gy IR, and fixed over 8 h and stained for DNA comet tails (×20 magnification). **e** Quantification of **d**. Bars show mean ± SEM (*n* = 3). *indicates *p* < 0.05 by *t*-test

Clonogenic assays confirmed that NF-κB inhibition by DMAPT treatment synergizes with IR in NSCLC cell lines (Fig. 2a). At doses that inhibit IκBα phosphorylation, DMAPT radiosensitized NSCLC cells, e.g., decreased clonogenic survival to a greater extent than that expected from additive lethality of the combination, i.e., the product of the percentages of surviving cells following each individual treatment (Supplementary Fig. S4). Alternately, cells expressing the NF-κB super-repressor showed similar degrees of radiosensitization as drug treatment. Importantly, addition of DMAPT to cells treated with the NF-κB super-repressor resulted in no further radiosensitization. This lack of additivity suggests that the effect of DMAPT on radiosensitivity is specific to its effect on the NF-κB pathway, as opposed to other potential effects of DMAPT such as generation of ROS.

To examine the impact of NF-κB inhibition on DNA DSB repair, the ability of NSCLC cells to resolve DSB-induced γ-H2AX foci^37^ was assessed by immunofluorescence. Following IR, there was rapid induction of γ-H2AX foci in all conditions. However, by 24 h post IR, a significant increase in persistence of γ-H2AX foci was observed in A549 cells treated with DMAPT that was not observed in cells treated with vehicle (Fig. 2b, c). To confirm that NF-κB inhibition, as opposed to other potential effects of DMAPT, results in radiosensitization, γ-H2AX foci were assessed in cells transfected with the NF-κB super-repressor. NF-κB inhibition with the super-repressor resulted in similar persistence of γ-H2AX foci as drug treatment. DMAPT treatment of cells transfected with the NF-κB super-repressor resulted in no further radiosensitization. This further suggested that DMAPT-induced DSB repair deficiency is specifically mediated by its effect on the NF-κB pathway.

To determine whether the increased accumulation of DSBs in DMAPT-treated cells was a result of increased DNA damage or a product of decreased DNA repair (persistence of damage), we quantified DNA damage accumulation via comet assay. About 1 h after 40 Gy IR treatment, NSCLC cells treated with DMAPT exhibited similar amounts of DNA damage assessed by olive tail moment compared to vehicle-treated cells (Fig. 2e; Supplementary Fig. S5). However, cells treated with DMAPT exhibited significantly larger olive tail moments 4 and 8 h following IR when compared to vehicle-treated cells. This suggests that DMAPT-induced NF-κB inhibition results in DSB accumulation by increasing the persistence of DNA damage.

### DMAPT reduces HR in NSCLC cells by downregulating expression and IR-induced focus formation of key HR proteins

**Fig. 3:**
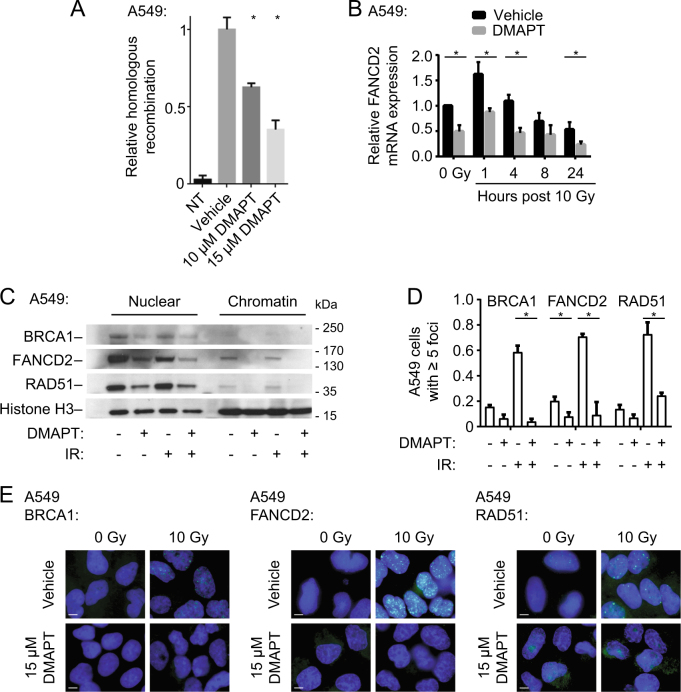
DMAPT reduces HR in NSCLC cells by downregulating expression and IR-induced focus formation of key HR proteins **a** HR measured by DR-GFP reporter flow cytometry assay in A549 cells treated with 0–15 µM DMAPT. A549 DR-GFP cells were treated with DMAPT for 24 h following transfection of an I-*SceI* plasmid. The percentages of GFP-positive cells were analyzed by flow cytometry. A549 cells without the DR-GFP integrant were used to measure background signal. All bars show mean ± SD (*n* = 3). * indicates *p* < 0.05 by *t*-test in pairwise comparisons to vehicle. **b** qPCR analysis of FANCD2 gene expression following 15 µM DMAPT and/or 10 Gy IR treatment in A549 cells. mRNA was collected at different time points up to 24 h post IR. Bars show mean ± SD (*n* = 3). **c** Western immunoblotting of nuclear and chromatin fractions of BRCA1, FANCD2, and RAD51 in A549 cells following DMAPT treatment and/or IR. Histone H3 was used as a loading control. **d** Quantification of BRCA1, FANCD2, and RAD51 focus formation via immunofluorescence of A549 cells incubated with or without DMAPT for 24 h, irradiated ±10 Gy and then fixed 6 h later. Bars show mean ± SD (*n* = 3). **e** Representative images of **d**. Results in NCI-H460 and NCI-H1299 cells are shown in Supplementary Fig. S4. Scale bar = 10 µm. *indicates *p* < 0.05 by *t*-test

Given the persistence of γ-H2AX foci and a previously characterized role of NF-κB signaling in the expression of FA/HR genes^22,23^, we hypothesized that DMAPT-induced impairment of DNA DSB repair may be a result of abrogation of the HR pathway. To quantify HR proficiency, the DR-GFP reporter assay, a fluorescence-based measurement of HR-mediated repair at a single I-*SceI*-induced DSB, was used^38^. A549 cells harboring a single integrant of the DR-GFP reporter were incubated with vehicle or DMAPT following induction of I-*SceI*-induced DSB. Cells treated with DMAPT exhibited significant dose-dependent decreases in HR activity compared to control (Fig. 3a). HR activity was also reduced by blocking NF-κB activation via the NF-κB super-repressor as a positive control.

We previously observed that defects in HR coincided with reduction of NF-κB-mediated expression of HR/FA genes^23^. In keeping with this, DMAPT treatment reduced *FANCD2* expression, both prior to and following 10 Gy irradiation, compared to control (Fig. 3b). Reduction of FANCD2 at the protein level was confirmed by western immunoblotting of whole-cell lysates (Supplementary Fig. S6). Decreases in expression of FANCD2, as well as BRCA1 and RAD51, were observed at the protein level in both nuclear and chromatin fractions (Fig. 3c).

To validate the observed HR defect, we assessed functional biomarkers of HR activity. BRCA1, FANCD2, and RAD51 foci are readily observed in response to DNA DSBs in HR-competent cells and the inability of these proteins to form nuclear foci is an indicator of HR deficiency^39^. Consistent with the reduction in BRCA1, FANCD2, and RAD51 protein expression, there were statistically significant decreases in the percentages of A549 (Fig. 3d, e), NCI-H460 and NCI-H1299 (Supplementary Fig. S7) cells that formed IR-induced foci of the three FA/HR proteins following DMAPT treatment.

### DMAPT reduces NHEJ in NSCLC cells by decreasing chromatin binding of key NHEJ proteins and inhibiting auto-phosphorylation of the DNA–PK complex

**Fig. 4:**
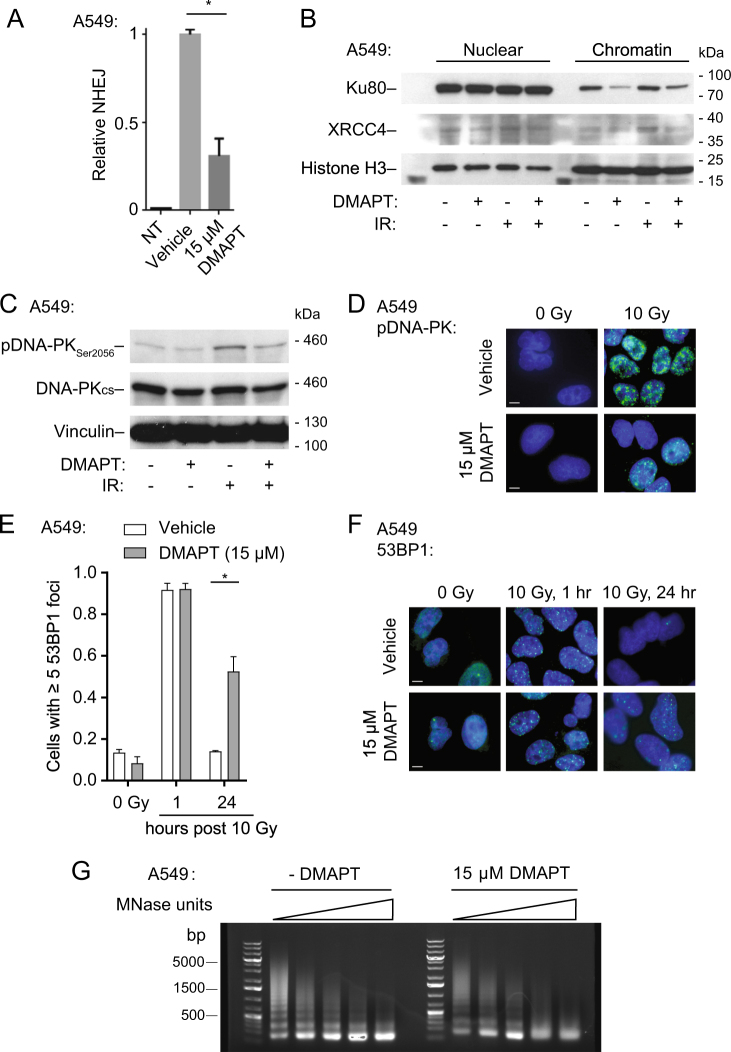
DMAPT reduces NHEJ activity in NSCLC cells by decreasing chromatin binding of key NHEJ proteins and inhibiting auto-phosphorylation of the DNA–PK complex **a** NHEJ measured by pEJ GFP reporter flow cytometry assay in DMAPT-treated cells. A549 pEJ cells were treated with DMAPT for 24 h following transfection of an I-*SceI* plasmid. The percentages of GFP-positive cells were analyzed by flow cytometry. Values were compared to non-transfected control. Bars show mean ± SD (*n* = 3). **b** Western immunoblotting of nuclear and chromatin fractions of Ku80 and XRCC4 in A549 cells following DMAPT treatment and/or IR. Cells were harvested 24 h following DMAPT treatment and 2 h following IR treatment. **c** Western immunoblot showing DNA-PK-Ser2056 phosphorylation levels following DMAPT treatment and/or 10 Gy IR in A549 cells. Cells were harvested 24 h following DMAPT treatment and 1 h following IR treatment. **d** Representative immunofluorescence images of DNA-PK-pS2056 focus formation following DMAPT and/or IR treatment in A549 cells. Cells were fixed 24 h following DMAPT treatment and 1 h following IR treatment. Results in NCI-H460 and NCI-H1299 cells are shown in Supplementary Fig. S6. Scale bar = 10 µm. **e** Quantification of 53BP1 foci in A549 cells treated with DMAPT and/or 10 Gy IR over a 24-h period. Bars show mean ± SD (*n* = 3). **f** Representative images of **e**. Scale bar = 10 µm. **g** DMAPT-induced changes in higher-order chromatin structure were assayed by chromatin digestion with increasing amounts of MNase followed by agarose gel electrophoresis. *indicates *p* < 0.05 by *t*-test

HR-competent cells primarily repair DNA DSBs through two pathways: HR and NHEJ. To clarify the specificity of DSB repair inhibition, we sought to assess NHEJ functionality following treatment with DMAPT. To assess NHEJ activity, the pEJ reporter system was used. The construct includes a premature stop codon flanked by two I-*SceI* sites, which when cut and repaired by NHEJ without the intervening sequence, restores GFP expression^40^. Following induction of I-*SceI* DSBs, a statistically significant decrease in NHEJ activity was observed in A549 cells treated with DMAPT compared to vehicle (Fig. 4a). To determine whether this phenomenon is specifically NF-κB mediated, the NF-κB super-repressor^32^ was expressed in A549 cells harboring the pEJ reporter. Expression of the NF-κB super-repressor decreased NHEJ activity, suggesting that NHEJ activity is mediated at least in part by NF-κB activity (Supplementary Fig. S8A).

To clarify the step in the NHEJ pathway that is impaired by DMAPT treatment, we assessed nuclear localization and chromatin binding of two proteins that mark key steps in NHEJ-mediated repair: Ku70/80, an upstream heterodimer important for initiation, recruitment and stabilization of other proteins, and XRCC4, a downstream component of the DNA ligase IV complex^41,42^. While significant changes in Ku70 messenger RNA (mRNA) levels in A549 whole-cell extracts (Supplementary Fig. S8B) or in Ku80 protein levels in A549 nuclear extracts (Supplementary Fig. S8C) could not be observed, there was a reduction in chromatin-bound Ku80 in DMAPT-treated cells, independent of irradiation (Fig. 4b). Further, XRCC4 protein levels were reduced in chromatin fractions of DMAPT-treated cells (Fig. 4b). DMAPT reduced IR-induced DNA-PK auto-phosphorylation and activation at Ser2056 (Fig. 4c) and pDNA-PK_S2056_ nuclear focus formation in A549 cells (Fig. 4d). Similar reductions in pDNA-PK_S2056_ nuclear focus formation were observed in NCI-H460 and NCI-H1299 cells (Supplementary Fig. S9). 53BP1 resolution after IR-induced DSBs was assessed as a biomarker of completed NHEJ^43,44^. Both DMAPT- and vehicle-treated A549 cells showed induction of 53BP1 nuclear foci 1 h after IR (Fig. 4e, f). However, 24 h post IR, cells treated with vehicle showed resolution of 53BP1 foci, while cells treated with DMAPT showed significant persistence of unresolved 53BP1 foci.

The DMAPT-induced decrease in binding affinity of vital NHEJ proteins early in the repair pathway suggest possible chromatin remodeling effects. To investigate this hypothesis, micrococcal nuclease (MNase) chromatin digestion assays were performed to assess changes in chromatin structure following DMAPT exposure. Chromatin from untreated cells showed laddering corresponding to incomplete chromatin digestion to single nucleosome-associated DNA fragments (Fig. 4g). In contrast, chromatin from DMAPT-treated cells showed loss of laddering corresponding to more complete digestion to single nucleosome-associated DNA fragments. At higher MNase concentrations, there was partial digestion of the mono-nucleosome fragments. These results show that accessibility to MNase digestion is modified by DMAPT treatment suggesting changes in higher-order chromatin structure.

## Discussion

The ability to repair DNA DSBs is critical to cellular survival following IR. In the setting of cancer therapy, it has been demonstrated that impairing the repair of DSBs is a potent mechanism to enhance the efficacy of RT^45^. DMAPT is an NF-κB inhibitor that has been observed to radiosensitize NSCLC cells^26^; however, the mechanism by which it does so, i.e., how it impairs repair of DNA DSBs, remained unknown. For the first time, we showed that DMAPT treatment impairs DNA DSB repair by causing a defect in both HR and NHEJ, which may be driven by decreased transcription of FA/HR genes and changes in chromatin structure that may impact DNA repair protein binding, respectively. This is in contrast to prior speculation that radiosensitization by DMAPT may be due to effects on cell cycle distribution^26,27^, which could not be reproducibly observed across cell lines in these prior studies or our studies. We showed via comet assays that DMAPT does not increase the generation of DNA DSBs but rather impairs their repair.

DMAPT has been shown to act on other pathways besides NF-κB, including glutathione/ROS, c-Jun, etc^46^. We demonstrated that radiosensitization by DMAPT is indeed primarily through inhibition of the NF-κB pathway as opposed to other potential effects, by showing that DMAPT had no further effects in cells expressing an NF-κB super-repressor. This included radiosensitization observed with clonogenic survival assays and persistence of γ-H2AX foci. If DMAPT radiosensitization were due in part to ROS, we would have expected to see additive effects in combination with the NF-κB super-repressor. We opted for this approach to show that it is NF-κB inhibition that results in radiosensitization by DMAPT, since attempts to block its effects on ROS, e.g., by using an antioxidant such as *N*-acetylcysteine, could also inhibit its effects on the NF-κB pathway by blocking sesquiterpene lactone alkylation of the Cys-38 residue of p65^47^.

Given an accepted role of NF-κB in the regulation of HR^22,48^, we initially investigated whether DMAPT impairs HR function. We directly observed that DMAPT impairs HR efficiency using a GFP-based reporter system. Assessment of a panel of HR biomarkers indicated a profound defect in IR-induced BRCA1, FANCD2, and RAD51 nuclear focus formation. The ability of these proteins to form DNA damage-induced foci is considered to be an appropriate marker for HR proficiency^12^. As HR is a cell cycle-dependent process, and previous data suggested that DMAPT may impair cell cycle progression^26^, we assessed the cell cycle distribution of multiple NSCLC cell lines following DMAPT and/or IR. No cell cycle defect was induced solely by DMAPT. Therefore, we concluded that the reduction in HR efficiency in our experiments was not the result of alterations in cell cycle distribution.

Our findings showed that NF-κB inhibition reduces FANCD2 mRNA levels, presumably by blocking NF-κB promoter binding, given the presence of promoter binding sites^22^. This in turn appeared to reduce FANCD2 protein availability for recruitment to DNA damage-induced foci. This mechanism is consistent with previous findings using different inhibitors^23^. However, other mechanisms are conceivable. For example, decreases in levels of HR proteins may be due to their destabilization and degradation^49–51^. Since NF-κB is a transcription factor, it seems most likely that DMAPT regulates HR at the mRNA level, however it remains possible that NF-κB regulates HR through additional mechanisms including at the protein level.

Interestingly, NF-κB signaling also appears to be implicated in NHEJ regulation. We explored the impact of NF-κB inhibition on NHEJ by examining critical biomarkers of the pathway. The defect in NHEJ was mediated in part by a reduction in DNA-PK activation-dependent auto-phosphorylation. DMAPT treatment did not appear to impact Ku mRNA expression. However, it decreased Ku chromatin association, and so DMAPT may inhibit NHEJ by limiting formation of the DNA-PK holoenzyme, which consists of the DNA-PK catalytic subunit and Ku^52^. Initial experiments assessing a possible role in modification of chromatin protein binding affinity suggested that DMAPT may be altering higher-order chromatin structure. These changes may be the driving factor behind decreased Ku chromatin binding and therefore decreased assembly and activation of the DNA-PK holoenzyme. Studies aimed at understanding the biochemical mechanism underlying DMAPT-induced modifications of chromatin affinity are needed to characterize further the role of NF-κB signaling in NHEJ.

Our study raises additional questions for further investigation. Genetically engineered mouse models that harbor single lung tumor nodules that can be targeted by conformal RT^53^ may be used to predict, which tumor genotypes may respond best to combined therapy with DMAPT and IR. For example, while KRAS mutant tumors frequently demonstrate poor responses to conventional therapies^54^, there is evidence that therapeutic resistance in KRAS mutant tumors may be driven in part by NF-κB^55,56^. Therefore, it is possible that NF-κB inhibitors may be particularly effective at improving the efficacy of DNA-damaging agents in KRAS-driven tumors. This may explain the excellent radiosensitization of KRAS mutant A549 xenografts by DMAPT without observable systemic toxicity in mice. As another example, others showed that DMAPT may selectively radiosensitize prostate tumors in transgenic mice compared to healthy tissues^57^. Identifying these settings will be crucial for establishing DMAPT as an agent that improves the therapeutic index.

Our study supports further development of DMAPT as a NSCLC radiosensitizer. DMAPT blocks DNA DSB repair by both HR and NHEJ. Our studies to understand further the mechanisms by which DMAPT blocks each DNA DSB repair pathway helped identify appropriate biomarkers for future studies. Combined, these data provide rationale for further preclinical and clinical evaluation of DMAPT.

## Materials and methods

### Cell lines and mice

Human NSCLC cell lines A549, NCI-H460, NCI-H1299, NCI-H23, NCI-H1915, NCI-H2087 and Calu-6, and human fetal fibroblast cell line MRC-5 were grown at 37 °C in a humidified 5% CO_2_ incubator. The NSCLC cell lines were grown in Roswell Park Memorial Institute (RPMI) 1640 medium (11875-093, Life Technologies, Grand Island, NY) and MRC-5 was grown in Eagle’s Minimum Essential Medium (30-2003, ATCC, Manassas, VA). Both media were supplemented with 10% fetal bovine serum (FBS, F2442, Sigma-Aldrich, St. Louis, MO) and 1 µg/ml Normocin (InvivoGen, San Diego, CA). All cell lines were obtained from and validated by short tandem repeat testing from the American Type Culture Collection (ATCC).

### Reagents

DMAPT (provided by Christopher Sweeney) was diluted in dimethyl sulfoxide (DMSO) (D8418, Sigma-Aldrich) to a concentration of 10 mM and stored at −20 °C for in vitro studies. Prior to use, DMAPT stocks were thawed and diluted in RPMI 1640 to a final concentration of 15 μM (for A549 and NCI-H460 cells) or 5 μM (for NCI-H1299 cells), unless stated otherwise. Bortezomib was diluted in DMSO to a concentration of 1 mM and stored at −80 °C for in vitro studies. Prior to use, bortezomib stocks were thawed and diluted in media to a final concentration of 50 nM. IR was given in vitro using an RS 2000 X-ray irradiator (Rad Source, Suwanee, GA) or Gammacell-40 Exactor Cs-137 irradiator (Best Theratronics, Ottawa, Ontario, Canada) with a central dose rate of 103 rad/min.

The following antibodies were used at the listed dilutions for western immunoblots: pIκBα_Ser32_ (14D4, Cell Signaling Technology, Danvers, MA, 1:500), IκBα (L35A5, Cell Signaling, 1:1000), BRCA1 (OP92, MilliporeSigma, Darmstadt, Germany, 1:500), FANCD2 (sc-20022, Santa Cruz Biotechnology, Santa Cruz, CA, 1∶200), RAD51 (PC130, MilliporeSigma, 1:1000), 53BP1 (sc-22760, Santa Cruz, 1:1000), pDNA-PK_Ser2056_ (ab18192, Abcam, Cambridge, MA, 1:200), DNA-PK_cs_ (sc-9051, Santa Cruz, 1:1000), Ku80 (PA5-17454, Thermo Fisher Scientific, Waltham, MA, 1:1000), XRCC4 (ab97351, Abcam, 1:1000), cleaved PARP1 (#5625, Cell Signaling, 1:1000), histone H3 (sc-10809, Santa Cruz, 1:1000), alpha-tubulin (DM1A) (sc-32293, Santa Cruz, 1:1000), vinculin (sc-25336, Santa Cruz, 1∶1000), anti-mouse (NA931v, GE Healthcare, UK; Little Chalfont, Buckinghamshire, UK, 1∶2500) and anti-rabbit (NA934v, GE Healthcare, UK, 1∶2500). The following antibodies were used at the listed dilutions for immunofluorescence: γ-H2AX (#05-636, MilliporeSigma, 1:1000), BRCA1 (sc-6954, Santa Cruz, 1∶50), FANCD2 (sc-20022, Santa Cruz, 1∶250), RAD51 (PC130, MilliporeSigma, 1:1000), pDNA-PK_Ser2056_ (ab18192, Abcam, 1:500), Alexa Fluor anti-mouse (4408S, Cell Signaling, 1∶2000), and anti-rabbit (4412S, Cell Signaling, 1∶2000).

### Cytotoxicity assays

For clonogenic assays, NSCLC cells were seeded into six-well tissue culture-treated dishes at 20% confluence. About 24 h after seeding, cells were transfected with/without pBabe-GFP-IKBalpha-mut (NF-κB super-repressor)^32^ and vehicle/DMAPT was initiated at the indicated doses. The pBabe-GFP-IKBalpha-mut plasmid was a gift from Dr. William Hahn. Following 23 h of treatment, DMAPT was replenished and cells were irradiated to the indicated doses 1 h later. About 6 h following irradiation, the cells were trypsinized, counted, and seeded in triplicate to generate individual colonies. At least 7 days later, the cells were fixed with methanol and stained with 1% crystal violet (C6158, Sigma-Aldrich). Thereafter, colonies of 50 or more cells were counted by eye. All results were normalized to cells treated with DMSO vehicle control.

For CellTiter-Glo assays, 96-well, white, clear bottom polystyrene tissue culture plates (#3903, Corning, Tewksbury, MA) were seeded with 1000 NSCLC cells in 200 μl media. After 24 h, media was replaced with media containing varying concentrations of DMSO/DMAPT. Cells were incubated for 24 h before luminescence was detected using the CellTiter-Glo Luminescent Cell Viability Assay (G7570, Promega, Madison, WI) per manufacturer’s protocol.

For the apoptosis assay, A549 cells were seeded in 10 cm plates at 20–40% confluence for 24 h. Media was then replaced with media containing DMSO or 15 μM DMAPT and cells were incubated for 24 h. Cells were then irradiated with 2 Gy and all conditions were collected 6 h post IR. Cleaved PARP1 was then assayed via western immunoblotting.

### Cell cycle profiling

NSCLC cells were seeded into six-well tissue culture-treated dishes at 20% confluence. About 24 h after seeding, cells were transfected with/without pBabe-GFP-IKBalpha-mut (NF-κB super-repressor)^32^ and vehicle/DMAPT treatment was initiated at the indicated doses. Following 23 h of treatment, the DMAPT was replenished and cells were irradiated to a dose of 10 Gy 1 h later. About 24 h after irradiation, cells were harvested, fixed with 70% ethanol in 15 ml conical polypropylene tubes and stored at −20 °C for at least 24 h prior to staining. On the day of staining, samples were harvested by centrifugation at 500 × *g* for 30 min, washed twice with PBS and incubated in propidium iodide staining solution (0.1% Triton X-100, 0.2 mg/ml RNase A, 20 μg/ml propidium iodide (P3566, Thermo Fisher)) for 15 min at 37 °C. Propidium iodide staining analysis was performed within 24 h using a BD LSRFortessa cell analyzer (BD Biosciences, San Jose, CA). Cell cycle analysis was performed using Modfit LT 5.0 software. Nonadherent dead cells and fragmented cells were excluded from analysis to assess cell cycle distribution of living cells.

### Western immunoblots and immunofluorescence

For whole-cell lysates, 1 ml ice-cold Pierce radioimmunoprecipitation assay (RIPA) buffer (89901, Thermo Fisher) with protease inhibitor cocktail (11 836 153 001, Roche Diagnostics, Mannheim, Germany) per 10^7^ cells was used. For chromatin fractionation, subcellular fractions were harvested using the Subcellular Protein Fractionation Kit for Cultured Cells (78840, Thermo Fisher) per the manufacturer’s protocol. SDS–PAGE was performed by adding 2× Laemmli buffer (161-0737, Bio-Rad) with 5% 2-mercaptoethanol (161-0701, Bio-Rad) to lysates and running samples in NuPAGE Novex 4–12% Bis-Tris Gels (NP0321, Thermo Fisher). Semi-dry transfer was performed using the Trans-Blot SD apparatus onto nitrocellulose membrane (Bio-Rad, Hercules, CA) per the manufacturer’s protocol. Western Lightning Plus enhanced chemiluminescence substrate (PerkinElmer, Waltham, MA) was used for visualization on Amersham Hyperfilm ECL (28906839, GE Healthcare, Pittsburgh, PA).

Band intensity was measured using the program ImageJ. Cleaved PARP band intensities were divided by the intensity of the respective vinculin loading controls. Ratios were normalized to the ratio of the untreated control condition.

For the pSer32 specificity assay, A549 cells were seeded into six-well tissue culture dishes and then transfected using Lipofectamine RNAiMAX Reagent (56532, Qiagen) with 25 nM IκBα siRNA (#1 = si00126826, #2 = si03114630, Qiagen) or AllStars Negative Control siRNA (102781, Qiagen). After 48 h, media was replaced with media containing 50 nM bortezomib for 1 h, prior to collection and western immunoblotting.

Immunofluorescence assays were performed as previously described^23^. Images were acquired for at least three replicates per condition at ×63 magnification using an Axio Imager Z1 fluorescence microscope with AxioCam MRc and MRm CCD cameras (Zeiss, Thornwood, NY). At least 150 cells per sample were imaged and scored blindly, with cells containing nuclei of five or more foci scored positive.

### Neutral comet assay

Cells were seeded into six-well plates at 20% confluence. DMAPT was initiated 24 h after seeding and refreshed 1 h prior to IR the following day. About 40 Gy IR was given with cells on ice, and cells were collected 1, 4, or 8 h after initiation of IR. The OxiSelect Comet Assay Kit (Cell Biolabs, San Diego, CA) was used per manufacturer’s protocol, and comets were visualized using an Axio Imager Z1 fluorescence microscope with an AxioCam MRm CCD camera (Zeiss, Thornwood, NY). Comet pictures were scored using ImageJ plugin Open Comet^58^.

### Homologous recombination and non-homologous end joining assays

A549 cells harboring single integrants of the phprtDRGFP (DR-GFP) or pEJ reporter were used for HR and NHEJ assays, respectively^23^. GFP reporter assays were performed as previously described^38,40^. Cells were transfected with pCBA*Sce* to induce DNA DSBs at I-*Sce*I sites introduced via phprtDRGFP or pEJ. About 2 (for pEJ) or 24 h (for phprtDRGFP) post-pCBA*Sce* transfection, cells were dosed with DMAPT at the indicated concentrations. About 48 h after pCBA*Sce* transfection, cells were harvested and analyzed for GFP expression using a BD LSRFortessa cell analyzer and FACSDiva software (BD Biosciences).

### Luciferase activity assay

A549 cells were plated in a 96-well plate at 1000 cells per well, transfected with the repressor or renilla luciferase control plasmid (pRL-CMV vector), and/or pBabe-GFP-IKBalpha-mut (NF-κB super-repressor)^32^. Media was replaced 6 h later and cells were then incubated with DMAPT or DMSO vehicle for 24 h before irradiated with 10 Gy. About 1 h after irradiation, luminescence was detected with the Dual-Luciferase Reporter (DLR) Assay System (E1910, Promega) and analyzed per manufacturer’s protocol.

### Quantitative PCR

Total RNA was isolated using the Purelink RNA Mini Kit (12183018A, Thermo Fisher) per manufacturer’s protocol. Complimentary DNA (cDNA) was synthesized using the High-Capacity cDNA Reverse Transcription Kit (4368814, Thermo Fisher) per manufacturer’s protocol. Quantitative RT-PCR was performed using *FANCD2* intron-spanning primers ACGGTGCTAGAGAGCTGCTT (forward) and TGTTCTCAGCACACTGGCAT(reverse) and*XRCC6* (Ku70) primers ATGGCAACTCCAGAGCAGGTG (forward) and AGTGCTTGGTGAGGGCTTCCA (reverse) (Eurofins MWG Operon, Huntsville, AL) with SYBR Green (4367659, Thermo Fisher) master mix. Data were normalized using *ACTB* primers TGAAGTGTGACGTGGACATC (forward) and GGAGGAGCAATGATCTTGAT (reverse).

### Chromatin relaxation assay

A549 cells were harvested in PBS and lysed with 10% glycerol, 0.5% NP40, 200 mM NaCl, 2 mM EDTA, 40 mM Tris-HCl pH 8.0 with protease inhibitors (Roche #11 873 580 001). Lysates were centrifuged at 10,000 × *g* for 20 min at 4 °C and pellets were washed with MNase buffer (20 mM Tris-HCl, pH 7.5, 100 mM KCl, 2 mM MgCl_2_, 1 mM CaCl_2_). The pellets were then digested with 25, 12.5, 6.25, 3.12, or 1.56 units of MNase (Roche #107 921), in MNase buffer for 10 min at 37 °C. The reaction was stopped with 9% SDS, 50 mM EDTA, pH 8, and proteins were digested with proteinase K (Qiagen #19131). After phenol–chloroform extraction and ethanol precipitation, the genomic DNA was diluted in TE buffer and analyzed on a 0.7% agarose gel.

### Statistics

All quantitative data were analyzed and graphed using Prism 6 (Graphpad Software, La Jolla, CA). To analyze the NF-κB luciferase assay, Tukey’s multiple comparison test was used, with a *p*-value < 0.05 viewed as significant. All other differences between treatment groups were determined via two-tailed Student’s *t* test, with a *p*-value < 0.05 viewed as significant.

## Electronic supplementary material

Supplementary Figures
