# Supplementary material for: NF-κB inhibition by dimethylaminoparthenolide radiosensitizes non-small-cell lung carcinoma by blocking DNA double-strand break repair
Source: Cell Death Discov. 2018 Feb 7;4:10. doi: 10.1038/s41420-017-0008-3 (PMC5841323; doi:10.1038/s41420-017-0008-3)
Supplement: Supplementary file 1 — Supplementary Figures [file 41420_2017_8_MOESM1_ESM.pdf]

SUPPLEMENTARY FIGURE LEGENDS

Figure S1. DMAPT IC50 values in a panel of NSCLC and fibroblast (MRC5) cell lines determined by Cell Titer-Glo cytotoxicity assays. Bars show mean ± SD (n=3).

Figure S2. (A) Western immunoblotting for pIκBα in NSCLC cells. 50 nM bortezomib was added one hour prior to cell harvest to block rapid proteasomal degradation of pIκBα so that it could be visualized. (B) Western immunoblotting for $pIkBa_{Ser32}$ in A549 cells treated with IκBa siRNAs to confirm antibody specificity.

Figure S3. Cell cycle analysis of (A) NCI-H460 and (B) NCI-H1299 cells following DMAPT treatment and/or 10 Gy IR. Cells were harvested 6 hours following IR or incubation without IR and analyzed by propidium iodide staining. DMAPT has no observable differences as a mono-therapy. Bars show mean ± SD (n=3).

Figure S4. Clonogenic assay quantification of the observed vs. expected survival of NSCLC cells treated with DMAPT at various doses of IR based on additive effects of each treatment, i.e., the product of the percentages of surviving cells following each individual treatment. Observed survival values lower than expected values indicate synergy beyond an additive effect. Bars show mean ± SD (n=3). * indicates $p < 0.05$ by t-test.

Figure S5. Comet assay images of (A) NCI-H460 and (B) NCI-H1299 cells treated with DMAPT and/or 40 Gy IR, and fixed over 8 hours and stained for DNA comet tails (20x magnification). (F) Quantification of (E). Bars show mean ± SEM (n=3). * indicates $p < 0.05$ by t-test.

Figure S6. Western immunoblotting for FANCD2 in A549 cells treated with 10 Gy IR ± 15 μM DMAPT and harvested 0-24 hours post-IR to assess decreases in total FANCD2 protein levels due to treatment, with tubulin as a loading control.

Figure S7. Quantification of BRCA1, FANCD2 and RAD51 focus formation in (A) NCI-H460 and (B) NCI-H1299 cells following DMAPT treatment and/or 10 Gy IR treatment. Bars show mean ± SD (n=3). (C-D) Representative images of (A-B). Scale bar = 10 μm. * indicates $p < 0.05$ by t-test.

Figure S8. (A) NHEJ measured by pEJ GFP reporter flow cytometry assay in A549 cells treated with 5 ng/ml TNFα to stimulate NF-κB or transfected with a non-phosphorylatable IκBα NF-κB super-repressor (NF-κB SR) to inhibit NF-κB signaling. Bars show mean ± SD (n=3). (B) Quantitative PCR for Ku70 expression normalized to ACTB in A549 cells treated with 10 Gy IR ± 15 μM DMAPT and harvested 0-24 hours post-IR. Bars show mean ± SD (n=3). (C) Western immunoblotting for Ku80 in A549 cells treated with 10 Gy IR ± 15 μM DMAPT and harvested 0-4 hours post-IR. * indicates $p < 0.05$ by t-test.

Figure S9. Representative immunofluorescence images of DNA-PK-pS2056 focus formation following DMAPT and/or IR treatment in (A) NCI-H460 and (B) NCI-H1299 cells. Cells were fixed 24 hours following DMAPT treatment and 1 hour following IR treatment. Scale bar = 10 μm.

Fig. S1

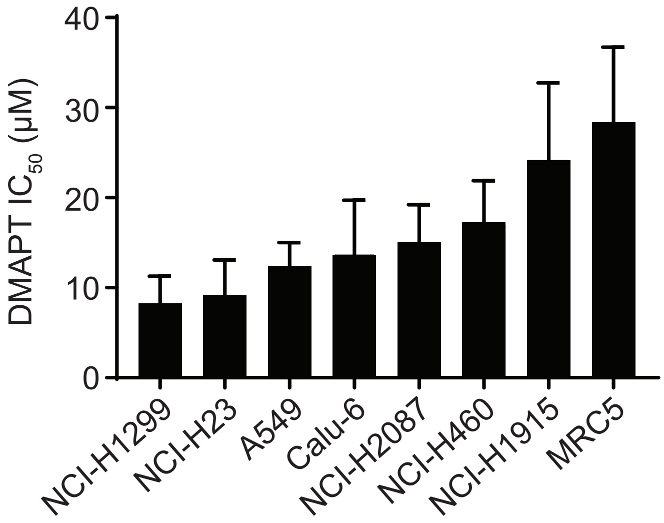

DMAPT IC50 (μM)
40
30
20
10
0
NCI-H1299
NCI-H23
A549
Calu-6
NCI-H2087
NCI-H460
NCI-H1915
MRC5

Fig. S2

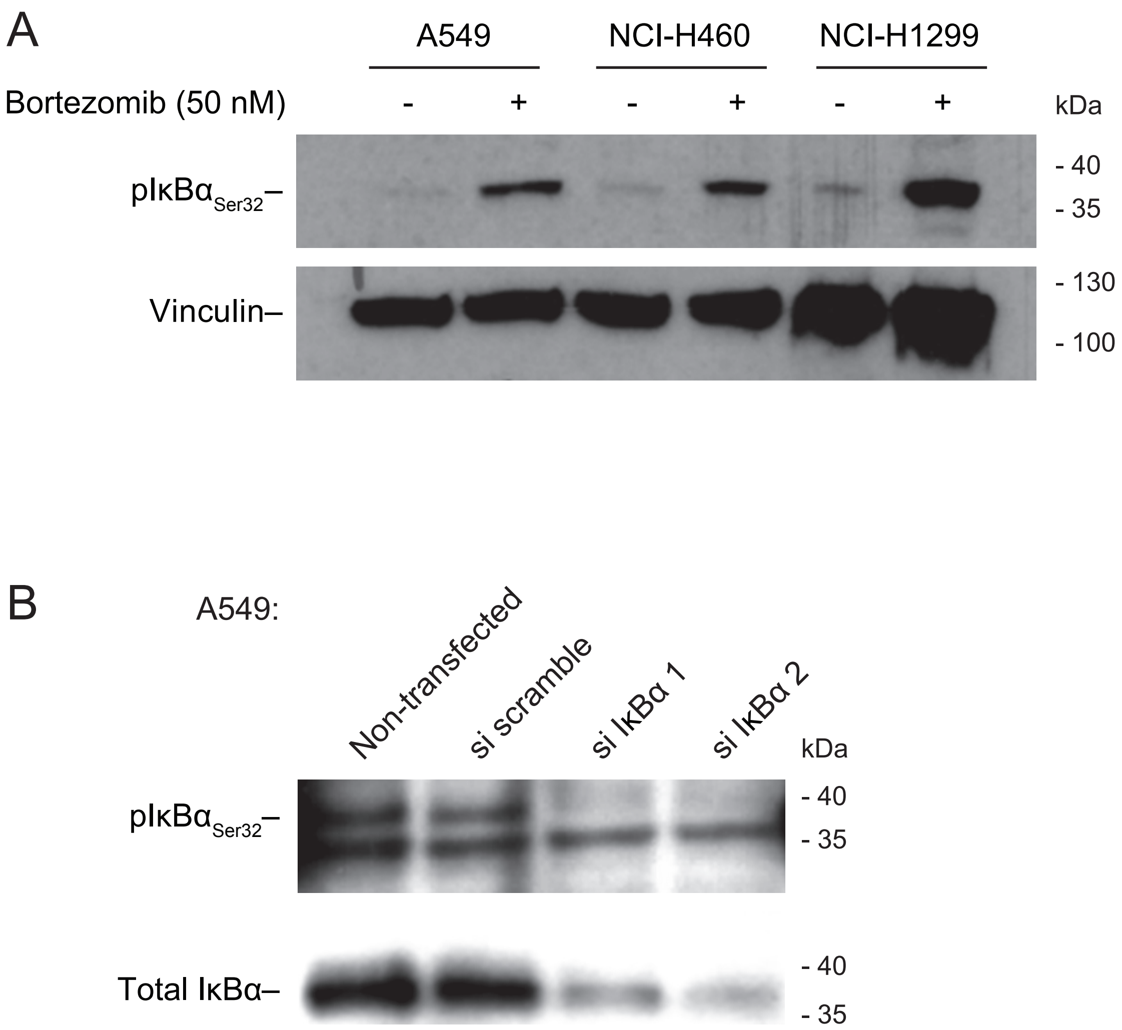
A
A549
NCI-H460
NCI-H1299
Bortezomib (50 nM)
-
+
-
+
-
+
kDa
pIκBα Ser32
- 40
- 35
Vinculin
- 130
- 100
B
A549:
Non-transfected
si scramble
si IκBα 1
si IκBα 2
kDa
pIκBα Ser32
- 40
- 35
Total IκBα
- 40
- 35

Fig. S3

A NCI-H460:

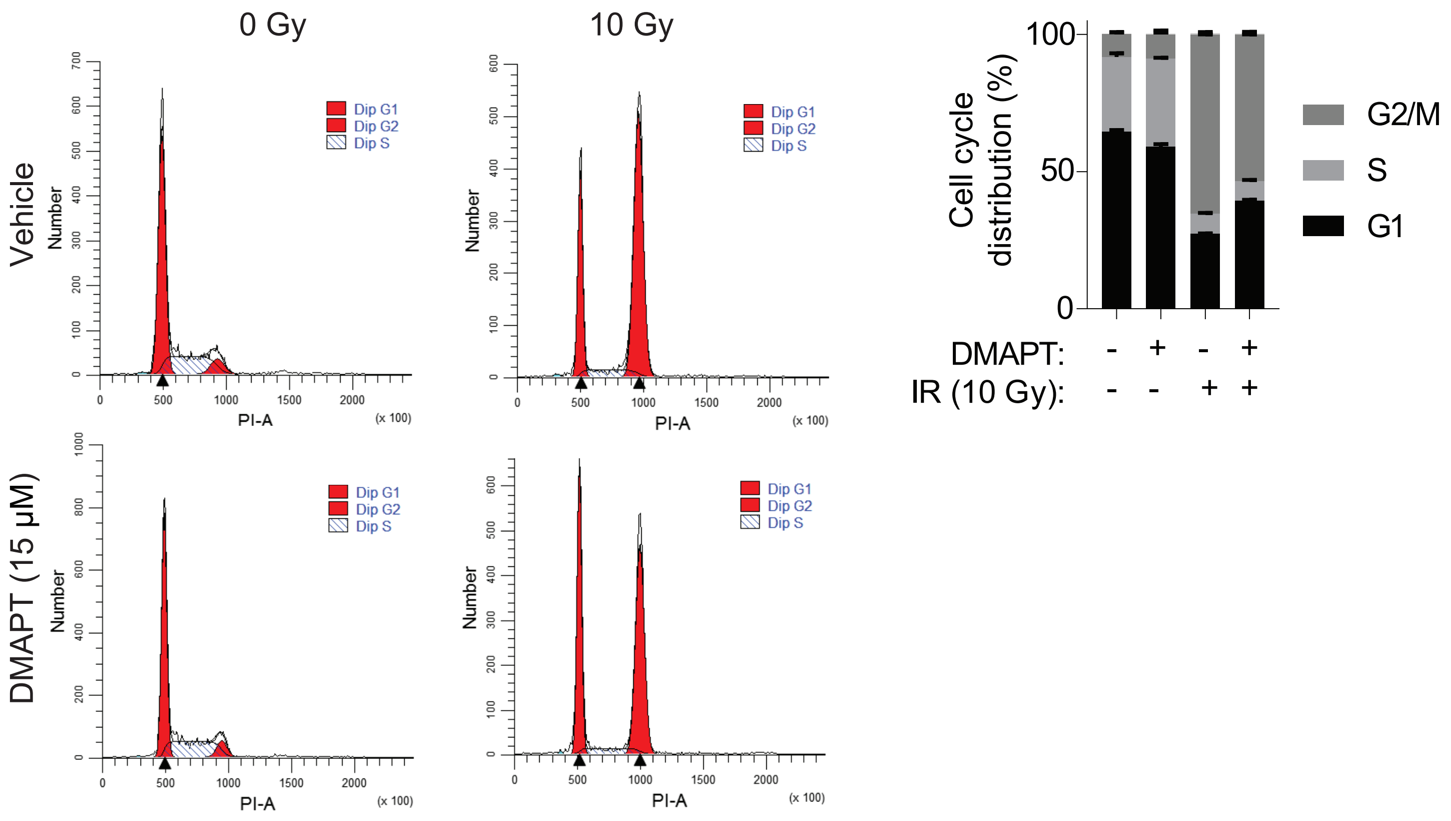

B NCI-H1299:

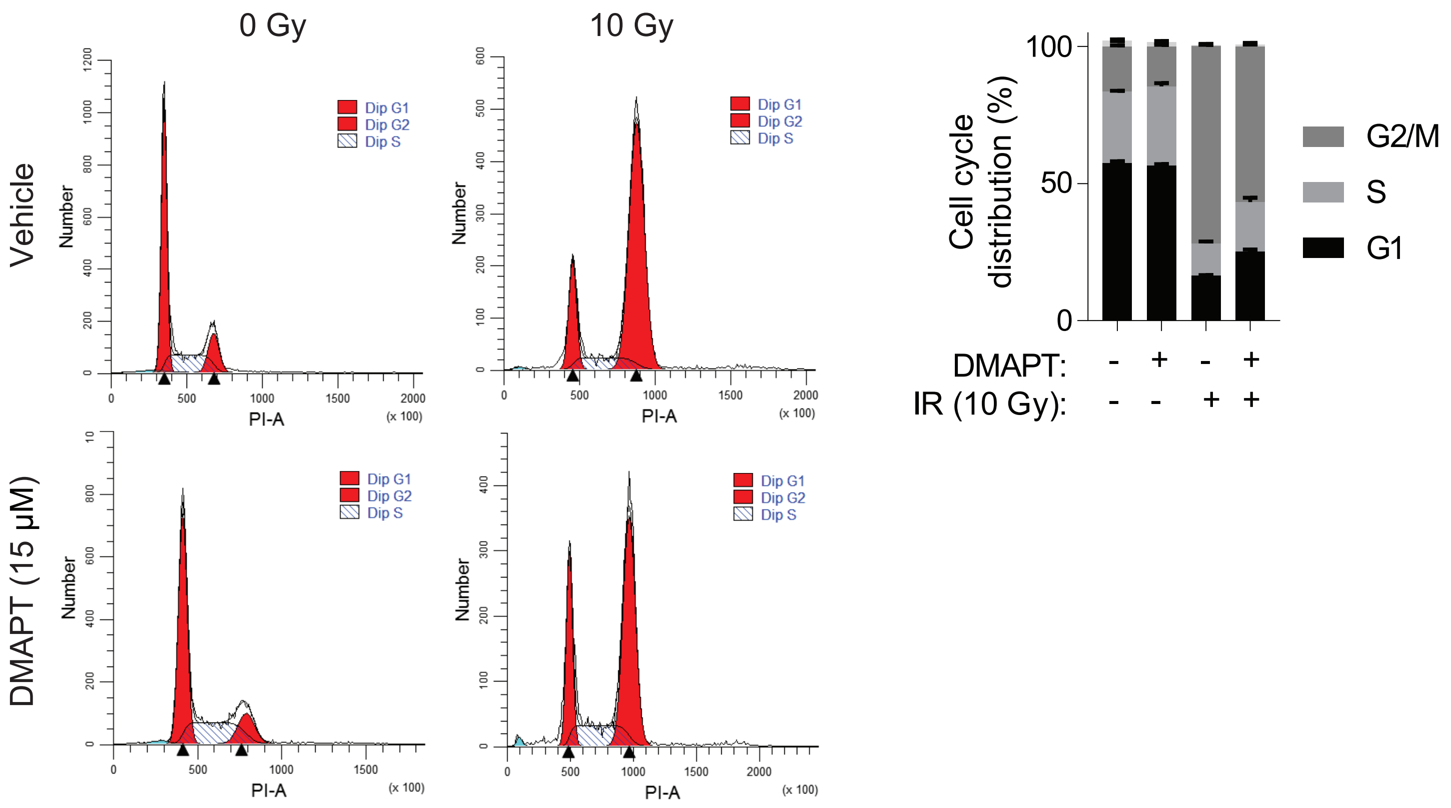

Fig. S4

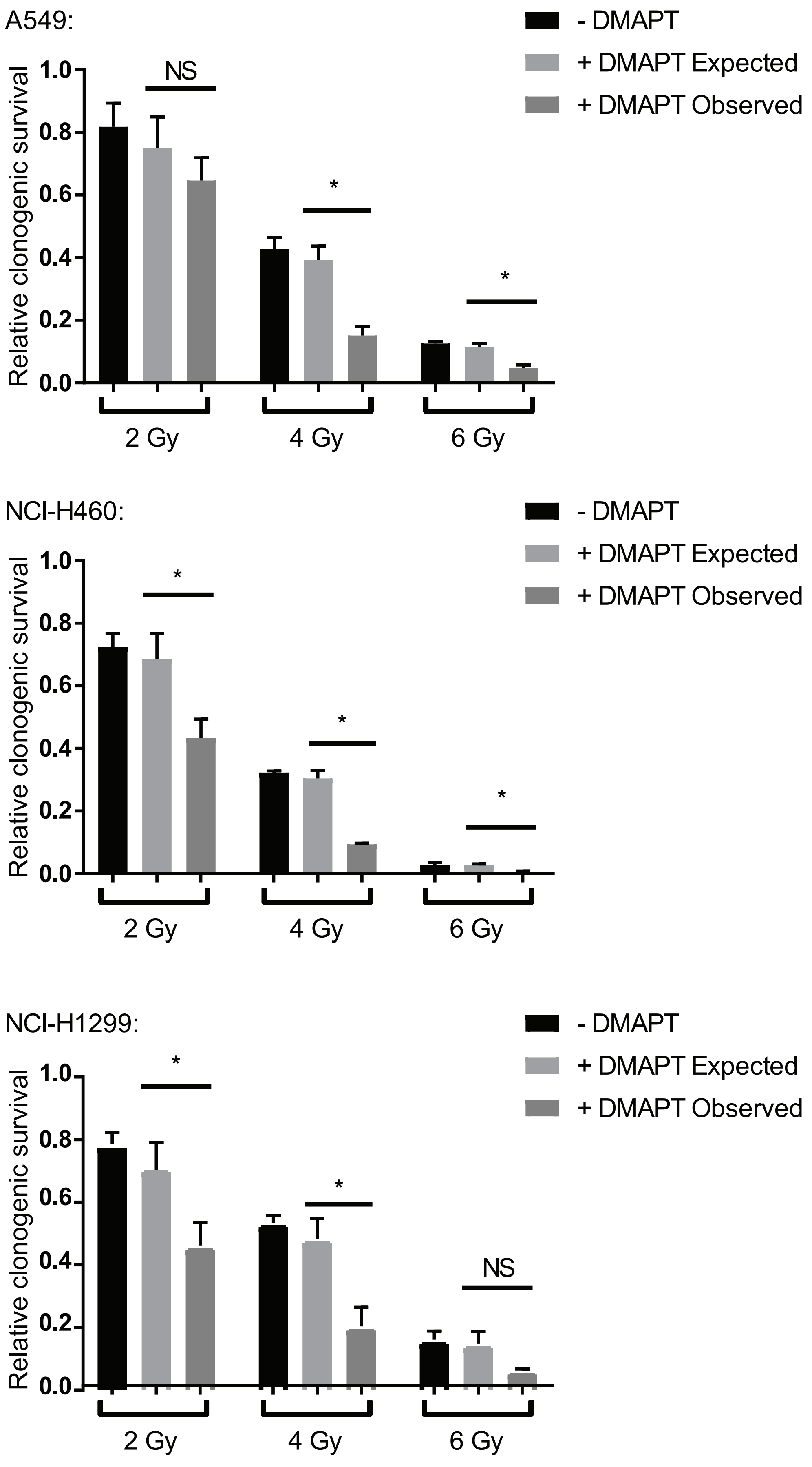
A549:
- DMAPT
+ DMAPT Expected
+ DMAPT Observed
Relative clonogenic survival
1.0
0.8
0.6
0.4
0.2
0.0
NS
*
*
2 Gy
4 Gy
6 Gy
NCI-H460:
- DMAPT
+ DMAPT Expected
+ DMAPT Observed
Relative clonogenic survival
1.0
0.8
0.6
0.4
0.2
0.0
*
*
*
2 Gy
4 Gy
6 Gy
NCI-H1299:
- DMAPT
+ DMAPT Expected
+ DMAPT Observed
Relative clonogenic survival
1.0
0.8
0.6
0.4
0.2
0.0
*
*
NS
2 Gy
4 Gy
6 Gy

Fig. S5

A

NCI-H460:

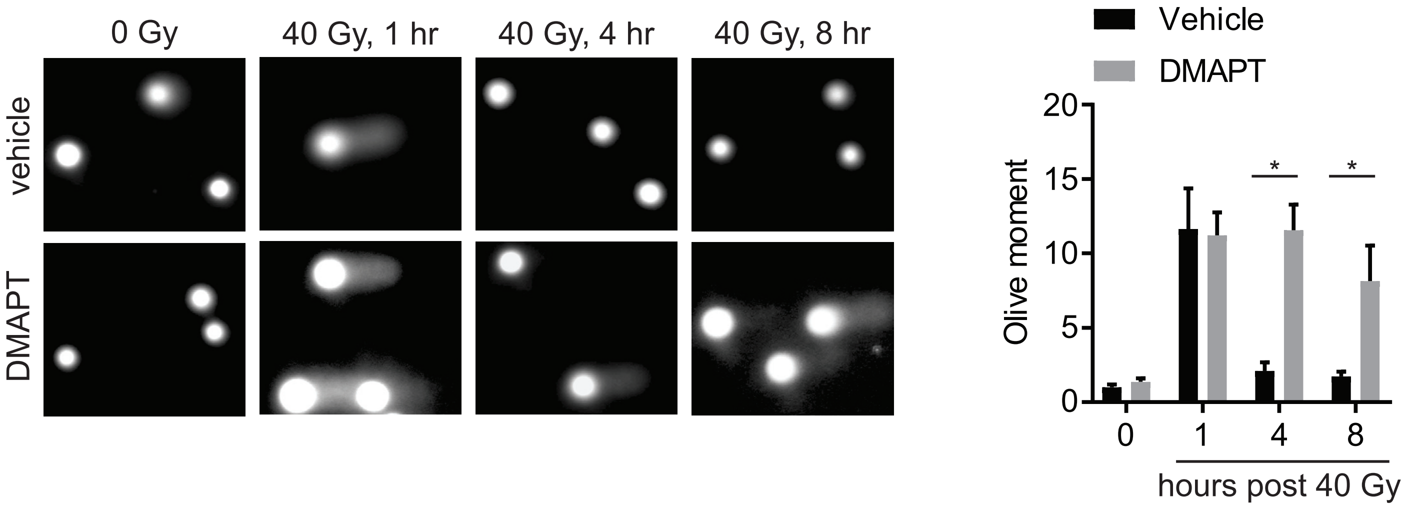

B

NCI-H1299:

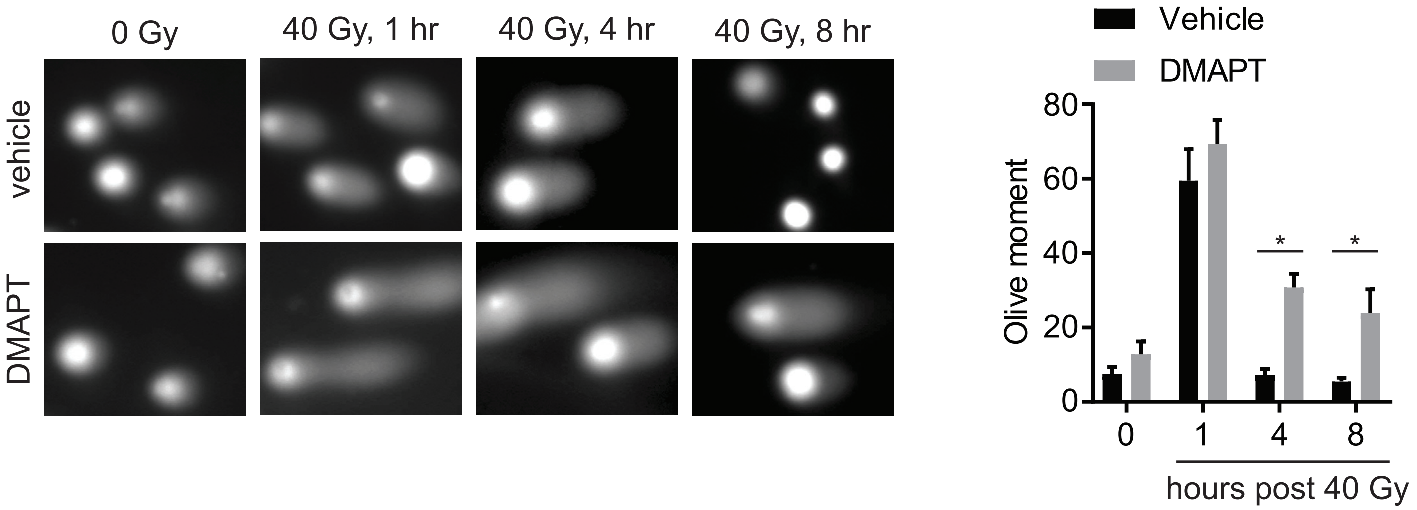

Fig. S6

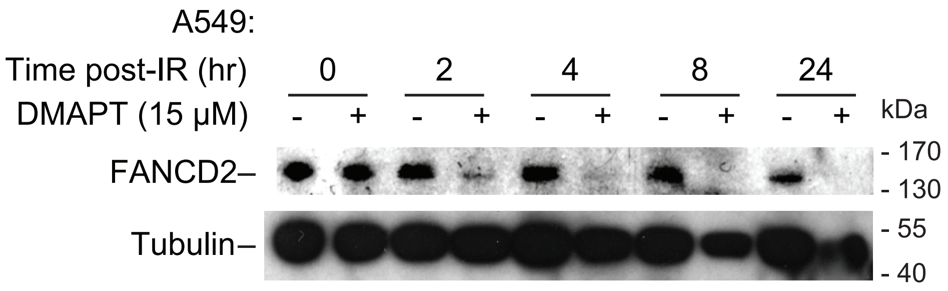
A549:
Time post-IR (hr)
0
2
4
8
24
DMAPT (15 μM)
- + - + - + - + - +
kDa
FANCD2–
- 170
- 130
Tubulin–
- 55
- 40

Fig. S7

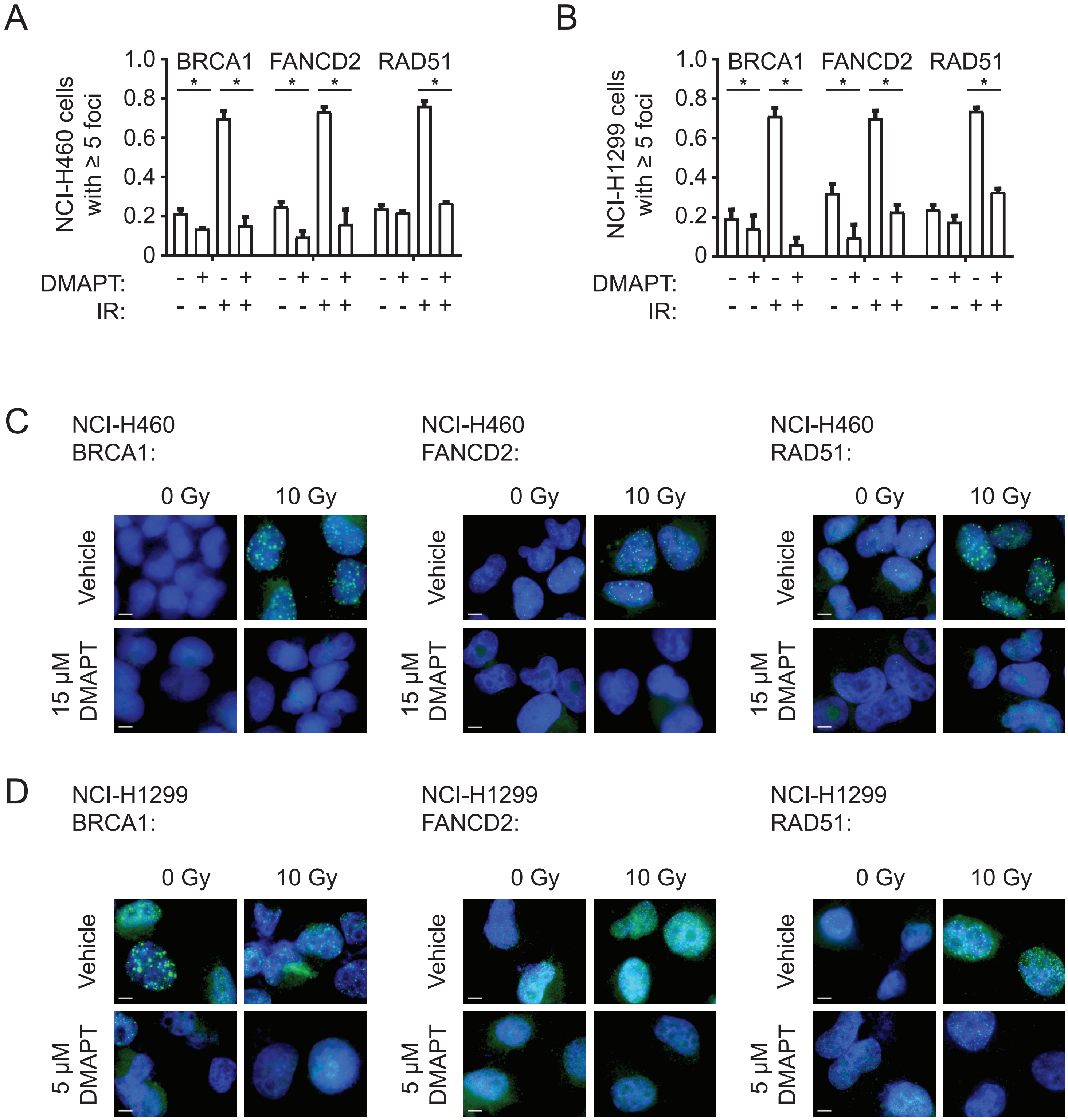
A
NCI-H460 cells with ≥ 5 foci
1.0
0.8
0.6
0.4
0.2
0
BRCA1
FANCD2
RAD51
*
DMAPT:
IR:
- + - +
- - + +
B
NCI-H1299 cells with ≥ 5 foci
1.0
0.8
0.6
0.4
0.2
0
BRCA1
FANCD2
RAD51
*
DMAPT:
IR:
- + - +
- - + +
C
NCI-H460 BRCA1:
NCI-H460 FANCD2:
NCI-H460 RAD51:
0 Gy
10 Gy
Vehicle
15 µM DMAPT
D
NCI-H1299 BRCA1:
NCI-H1299 FANCD2:
NCI-H1299 RAD51:
0 Gy
10 Gy
Vehicle
5 µM DMAPT

Fig. S8

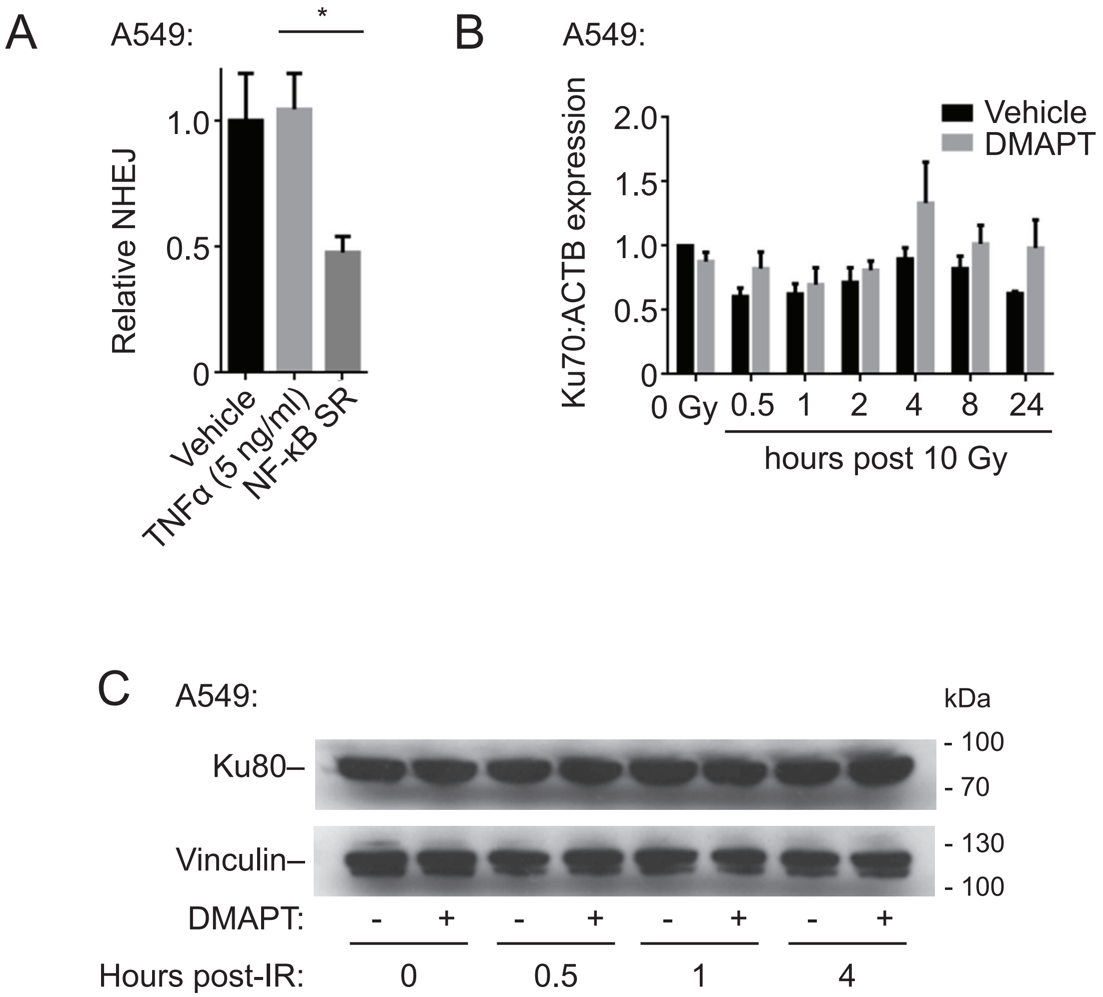

A
A549:
*
Relative NHEJ
1.0
0.5
0
Vehicle
TNFα (5 ng/ml)
NF-κB SR
B
A549:
Ku70:ACTB expression
2.0
1.5
1.0
0.5
0
Vehicle
DMAPT
0 Gy
0.5
1
2
4
8
24
hours post 10 Gy
C
A549:
kDa
Ku80–
- 100
- 70
Vinculin–
- 130
- 100
DMAPT: - + - + - + - +
Hours post-IR: 0 0.5 1 4

Fig. S9

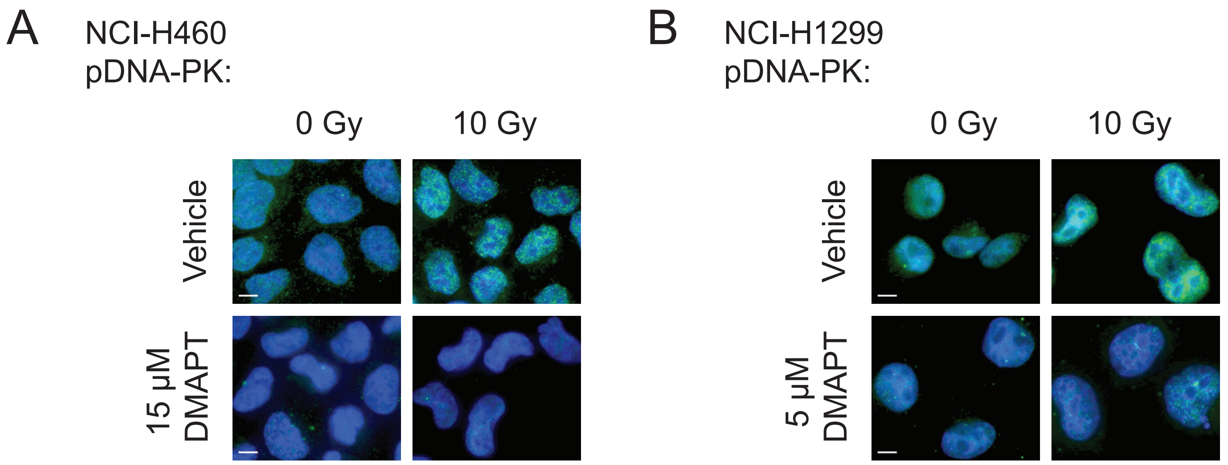
A
NCI-H460
pDNA-PK:
0 Gy
10 Gy
Vehicle
15 µM DMAPT
B
NCI-H1299
pDNA-PK:
0 Gy
10 Gy
Vehicle
5 µM DMAPT
